# Supplementary material for: Neural dynamics of the attentional blink revealed by encoding orientation selectivity during rapid visual presentation
Source: Nat Commun. 2020 Jan 23;11:434. doi: 10.1038/s41467-019-14107-z (PMC6978470; doi:10.1038/s41467-019-14107-z)
Supplement: Supplementary file 1 — Supplementary Information [file 41467_2019_14107_MOESM1_ESM.pdf]

Supplementary Information

Tang, et al., Neural dynamics of the attentional blink revealed by encoding orientation selectivity during rapid visual presentation

$$G(x) = A \exp - \frac{(x-\mu)^2}{2\sigma^2} + C$$

A = Gain
σ = Width
C = Baseline

μ = Centre orientation set to 90 deg

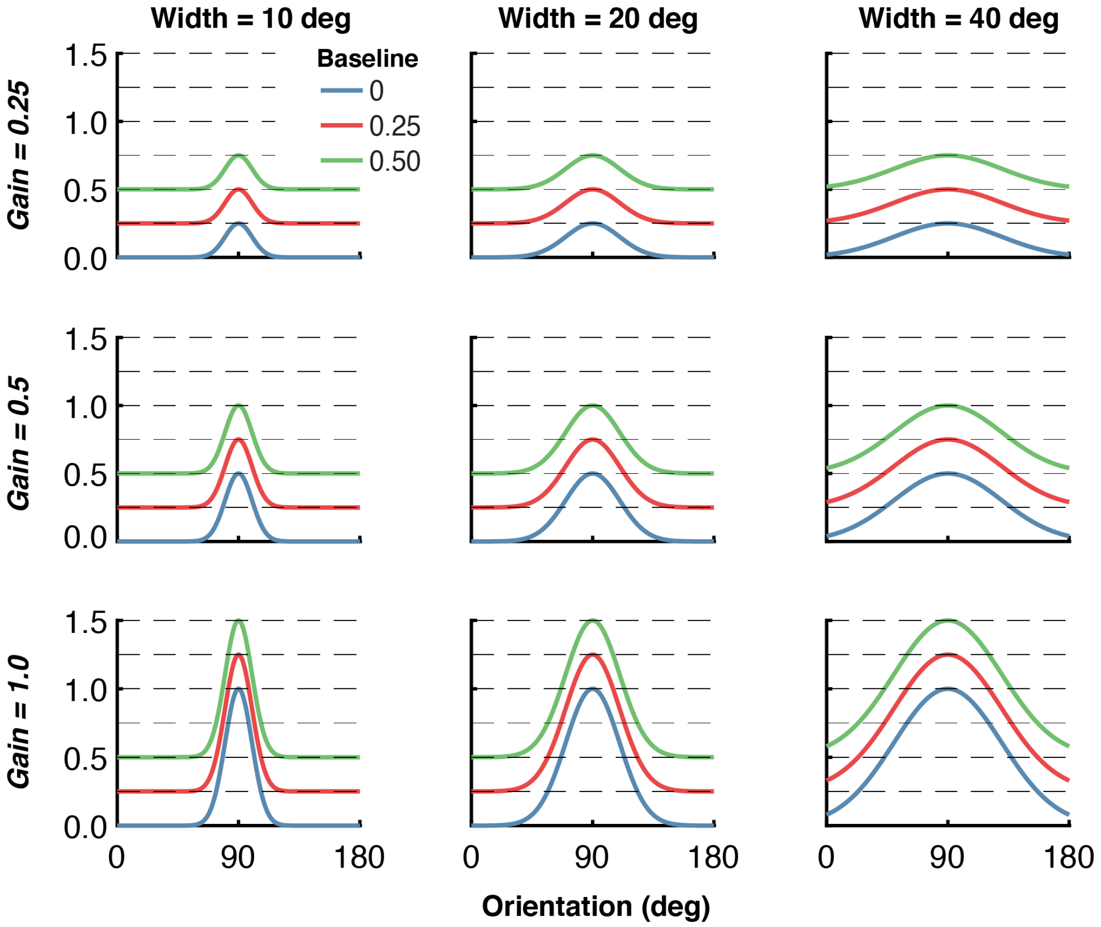

Supplementary Figure 1. Examples of the four-parameter Gaussians used to quantify behavioural orientation errors and forward encoding representations for orientation from EEG activity. The figure shows the effect of different parameter values on the shape of the resulting function. Each row has a different gain value, and each column has a different width parameter. Within each panel, the baseline value changes. The width parameter shows the precision (of either the behavioural responses or neural representations). The baseline parameter captures non-selective responses that are unrelated to the target. For the behavioural analysis, this reflects random guessing which would be distributed equally across all orientations; for the EEG analysis, it reflects overall, non-feature selective activity from the orientation encoding. For all panels, the centre orientation of the Gaussian is set to 90°. The figure highlights the independence of the parameters of the Gaussians. For instance, looking at the panels across a given row (where width varies, but gain is fixed) reveals that curves with the same baseline value have peaks at the same height. Inspecting any one panel shows that the baseline and gain parameters are independent, with the differences between the peaks of the curves being equal regardless of the baseline.

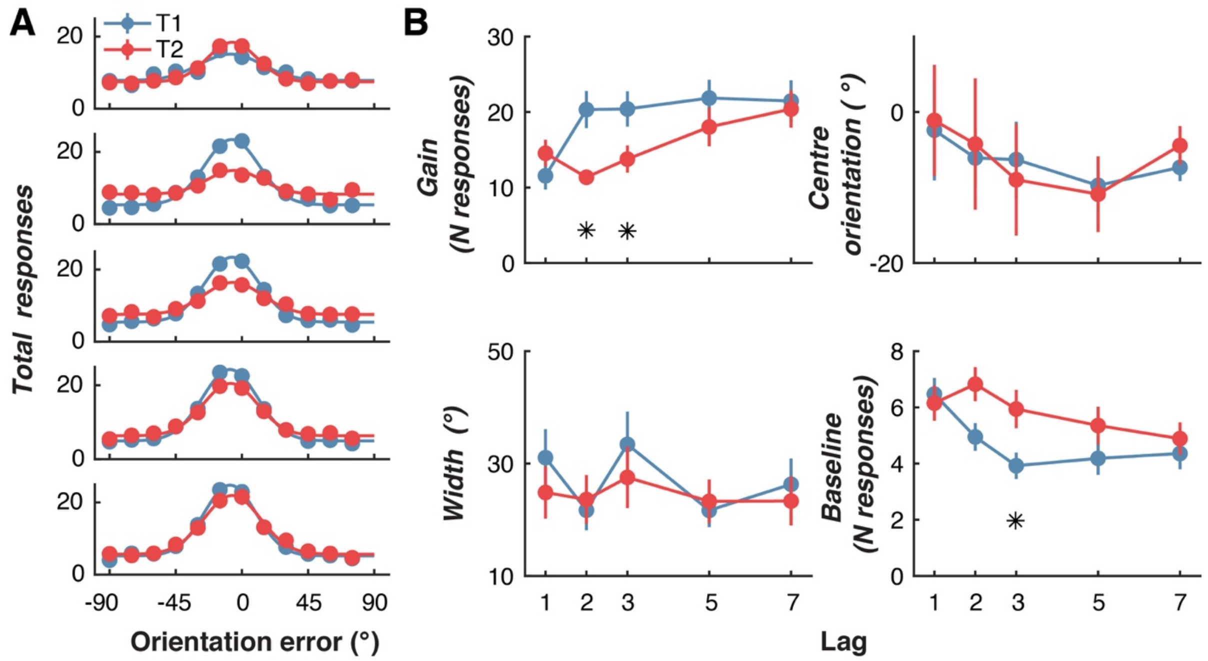

Supplementary Figure 2. Re-analysis of behavioural results in Experiment 1. In the original analysis (Figure 2), the responses were normalized to 1 for each participant to show the proportion of total responses in each orientation error bin. To confirm that this normalization did not bias the results, we re-ran the analysis but used the total number of responses (120 trials for each condition). **A.** The distribution of response errors (difference between presented and reported orientation) across participants for T1 and T2 for each Lag condition. Lines show fitted Gaussian functions. **B.** Quantified behavioural responses for the four parameters of the fitted Gaussian function for each participant. We used 2 (Target; T1,T2) x 5 (Lag; 1,2,3,5,7) within-subject ANOVAs to quantify the effect of the AB on the parameters of the fitted Gaussians. The gain parameter was affected by the factors of Target ($F(1,21)=8.97$, $p=0.007$, $\eta_p^2=0.30$) and Lag ($F(4,84)=11.78$, $p<.0001$, $\eta_p^2=0.36$), and there was a significant interaction between these factors ($F(4,84)=7.29$, $p<.0001$, $\eta_p^2=0.26$). By contrast, and consistent with the original analysis, for the width parameter there were no significant main effects of Target ($F(1,21)=0.54$, $p=0.47$, $\eta_p^2=0.02$) or Lag ($F(4,84)=1.08$, $p=0.37$, $\eta_p^2=0.05$), and no interaction($F(4,84)=0.60$, $p=0.66$, $\eta_p^2=0.03$). The baseline parameter, which reflects guessing of random orientations, was significantly affected by Target ($F(1,21)=8.72$, $p=0.008$, $\eta_p^2=0.29$) and Lag ($F(4,84)=3.54$, $p=0.01$, $\eta_p^2=0.14$). There was also a significant interaction between these factors ($F(4,84)=3.04$, $p=0.02$, $\eta_p^2=0.13$). Taken together, the results replicate those reported in the main analysis, and confirm that the process of normalization did not bias the outcomes. Asterisks indicate Bonferroni-corrected differences at $p < 0.05$. Error bars indicate ∓1 standard error of mean.

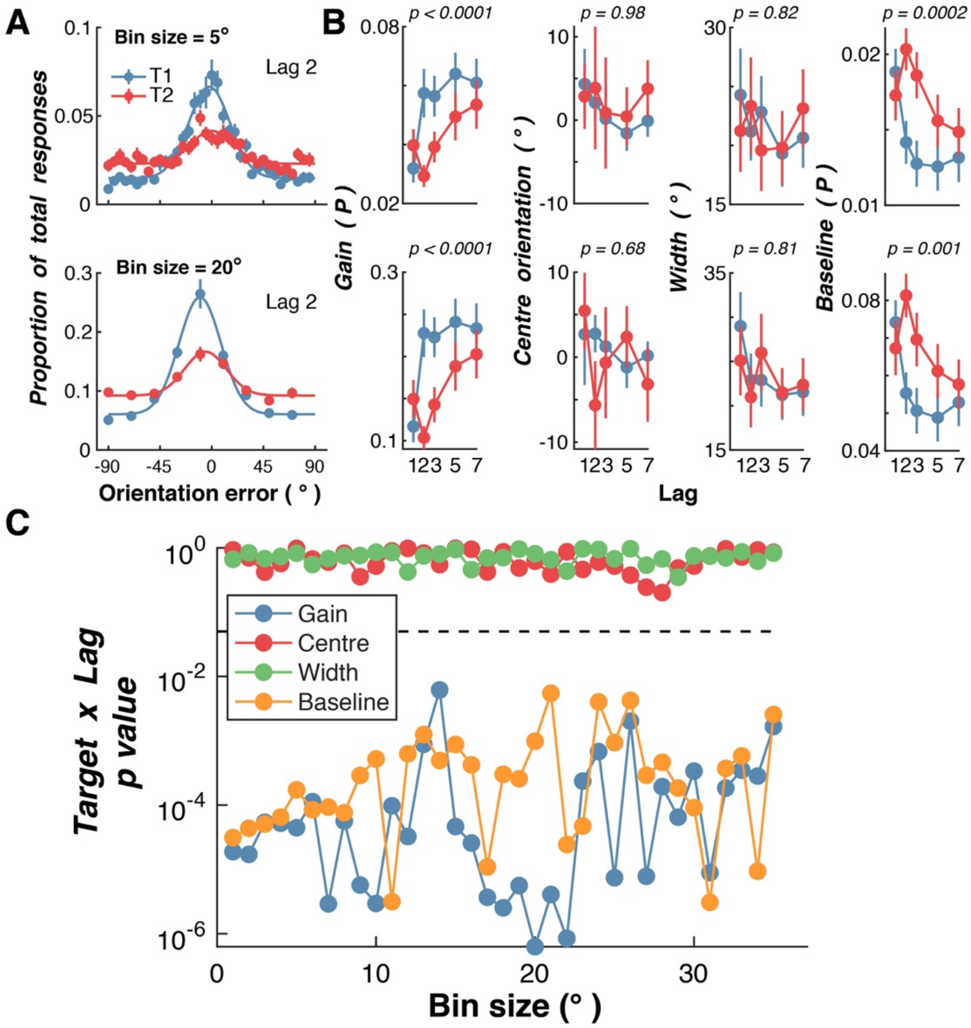

Supplementary Figure 3. Re-analysis of behavioural results from Experiment 1, with different bin sizes of orientation errors to generate the orientation error histogram. In the original analysis, a 15° bin size was used to group responses. **A.** Examples of Lag 2 histograms for two bin sizes (5° and 20°) for responses across participants. For the 5° bin size, orientation errors from -90° to -86° would be grouped together. By contrast, for a 20° bin, orientation errors from -90° to -71° would be grouped together. **B.** For each participant, Gaussians were fit to the resulting response function for each Lag and Target (T1 and T2). Here the fits are shown for 5° (top row) and 20° (bottom row) for the four parameters of the Gaussian. The p value is for the interaction term from the within-subjects ANOVA used in the original analysis, with factors of Lag (1,2,3,5,7) x Target (T1, T2). In the original analysis, as in the classic AB, a significant Lag x Target interaction shows that T2 accuracy is impaired at early Lags, whereas T1 accuracy is unaffected by Lag. **C.** P-values for the interaction term (Lag x Target) across a wide range of bin sizes for the four Gaussian parameters. The dotted line indicates p = 0.05. Note that the p values are displayed on a log axis. As in the original analysis, across this wide range of bin sizes, there was a clear AB effect on the gain and baseline parameters of the Gaussian, but no such effect on the width or centre orientation parameters.

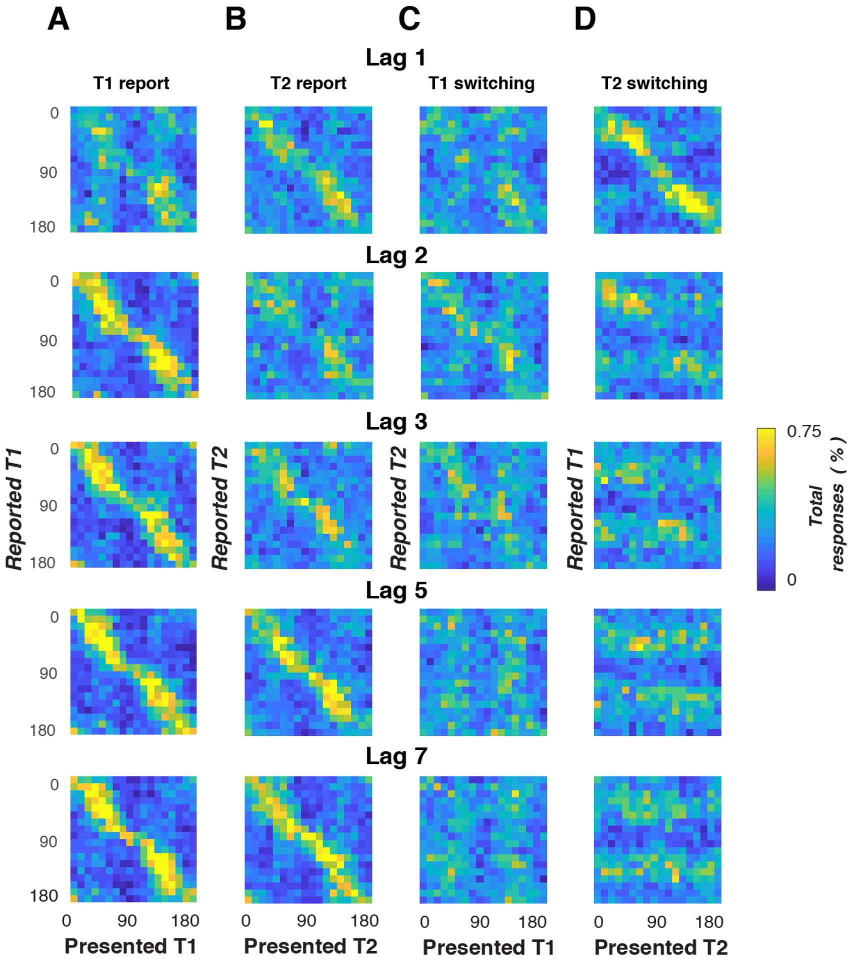

Supplementary Figure 4. Heatmaps of reported versus presented orientations across all participants in Experiment 1. These are analogous to confusion matrices for a continuous report task. Warmer colours indicate a greater proportion of responses. Note that participants were asked to report the targets in their presented order. Each row is a lag and each column shows a different comparison. **A**. Presented-T1 orientation against reported-T1 orientation. For Lags 2-7, there is a strong correspondence between presented and reported orientations, confirming that participants accurately reported T1 targets. **B**. The outcome of the same analysis, but for T2 targets. For these items, there was a strong correspondence between presented and reported orientations at Lags 1, 5 and 7, which decreases (i.e., more random) for items at Lags 2 and 3. **C**. T1 switching, where presented-T1 orientation is plotted against reported-T2 orientation. **D.** T2 switching, where presented-T2 orientation is plotted against reported-T1 orientation. Clear switching is evident only at Lag 1, where the orientation of the item presented at T2 is reported as the orientation of T1.

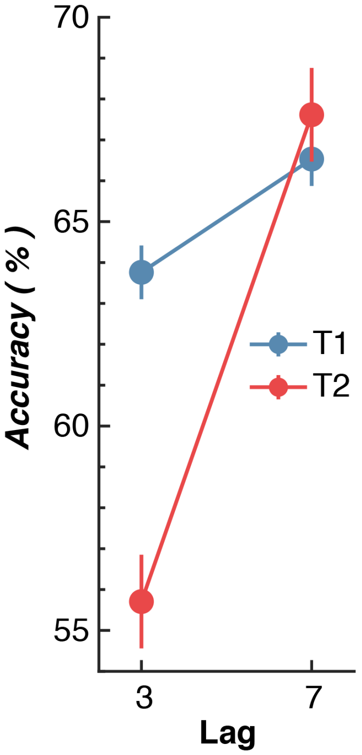

Supplementary Figure 5. Behavioural accuracy for the RSVP task in Experiment 2. Each response was scored as correct if the participant responded within ±30° of the presented orientation. Following conventional AB analysis procedures, T2 accuracy was scored using only those trials in which the T1 response was correct (T2lT1). Error bars show within-subject standard error.

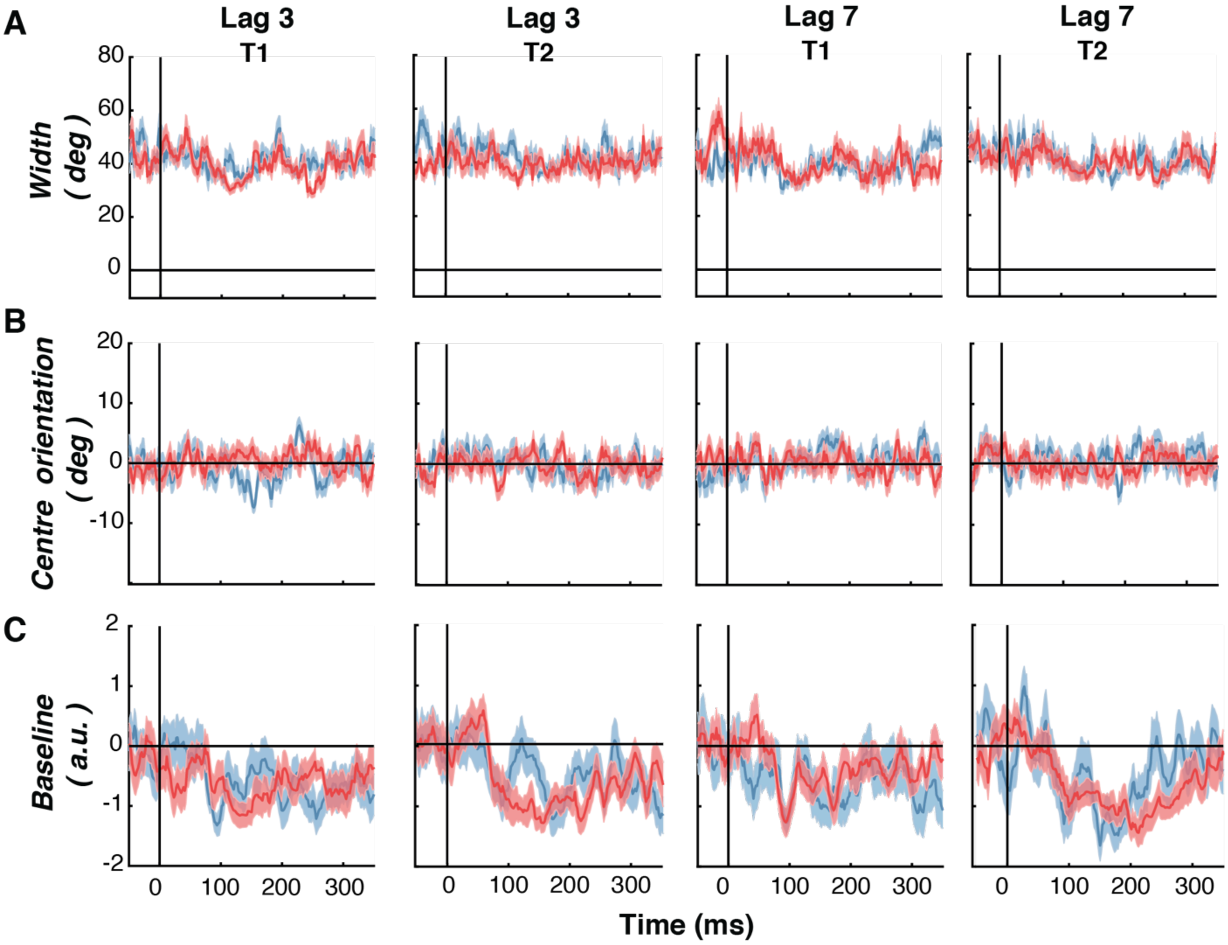

Supplementary Figure 6. Plots of three fitted parameters for the neural representations of feature-selective information over time for T1 and T2 items in Experiment 2. **A.** Time course of measured width of feature selectivity for T1 and T2 items, given by the width (standard deviation) of the fitted Gaussian. Trials were scored as correct if the participant's response was within 30° of the presented orientation. Only trials in which participants responded correctly to T1 were included in the analysis. **B.** Same as in panel **A** but for the centre orientation parameter. **C.** Same as in panel **A** but for the baseline parameter. For all panels, there were no significant differences between conditions (two-tailed cluster-permutation, alpha $p < 0.05$, cluster alpha $p < 0.05$, N permutations = 20,000). Shading indicates ∓1 standard error of mean.
