# Supplementary material for: Neural dynamics of the attentional blink revealed by encoding orientation selectivity during rapid visual presentation
Source: Nat Commun. 2020 Jan 23;11:434. doi: 10.1038/s41467-019-14107-z (PMC6978470; doi:10.1038/s41467-019-14107-z)
Supplement: Supplementary file 3 — Reporting Summary [file 41467_2019_14107_MOESM3_ESM.pdf]

natureresearch

Corresponding author(s): Matthew Tang

Last updated by author(s): Nov 16, 2019

Reporting Summary

Nature Research wishes to improve the reproducibility of the work that we publish. This form provides structure for consistency and transparency in reporting. For further information on Nature Research policies, see Authors & Referees and the Editorial Policy Checklist .

Statistics

For all statistical analyses, confirm that the following items are present in the figure legend, table legend, main text, or Methods section.

n/a	Confirmed	
☐	☒	The exact sample size (n) for each experimental group/condition, given as a discrete number and unit of measurement
☐	☒	A statement on whether measurements were taken from distinct samples or whether the same sample was measured repeatedly
☐	☒	The statistical test(s) used AND whether they are one- or two-sided<br>*Only common tests should be described solely by name; describe more complex techniques in the Methods section.*
☒	☐	A description of all covariates tested
☐	☒	A description of any assumptions or corrections, such as tests of normality and adjustment for multiple comparisons
☐	☒	A full description of the statistical parameters including central tendency (e.g. means) or other basic estimates (e.g. regression coefficient) AND variation (e.g. standard deviation) or associated estimates of uncertainty (e.g. confidence intervals)
☐	☒	For null hypothesis testing, the test statistic (e.g. F, t, r) with confidence intervals, effect sizes, degrees of freedom and P value noted<br>*Give P values as exact values whenever suitable.*
☒	☐	For Bayesian analysis, information on the choice of priors and Markov chain Monte Carlo settings
☒	☐	For hierarchical and complex designs, identification of the appropriate level for tests and full reporting of outcomes
☐	☒	Estimates of effect sizes (e.g. Cohen's d, Pearson's r), indicating how they were calculated

Our web collection on statistics for biologists contains articles on many of the points above.

Software and code

Policy information about availability of computer code

Data collection	PsychToolbox for MATLAB was used for stimulus presentation. EEG data were collected using a 64-channel BioSemi system.
Data analysis	All data were analyzed using MATLAB. The code is available at https://github.com/MatthewFTang/AttentionalBlinkForwardEncoding.

For manuscripts utilizing custom algorithms or software that are central to the research but not yet described in published literature, software must be made available to editors/reviewers. We strongly encourage code deposition in a community repository (e.g. GitHub). See the Nature Research guidelines for submitting code & software for further information.

Data

Policy information about availability of data

All manuscripts must include a data availability statement. This statement should provide the following information, where applicable:

- Accession codes, unique identifiers, or web links for publicly available datasets
- A list of figures that have associated raw data
- A description of any restrictions on data availability

The data is available at https://osf.io/f9g6h.

Field-specific reporting

Please select the one below that is the best fit for your research. If you are not sure, read the appropriate sections before making your selection.

☒ Life sciences ☐ Behavioural & social sciences ☐ Ecological, evolutionary & environmental sciences

For a reference copy of the document with all sections, see nature.com/documents/nr-reporting-summary-flat.pdf

Life sciences study design

All studies must disclose on these points even when the disclosure is negative.

Sample size	The participants were recruited from a paid participant pool which they self selected into after reading a brief description of the research. This would be a sample of convenience. No sample size calculation was performed prior to testing. The sample size was made to be the the larger than we have previously used to study to the Attentional Blink. Importantly, each subject completed 600 trials of testing which allowed us to reduce within-subject variably of the reported effects.
Data exclusions	No participants were excluded from the analysis.
Replication	All modelling was done on each subject's data with the effects replicated across the subjects. Statistical analysis determined whether these effects were consistent across subjects.
Randomization	The study used a fully-within subjects design so there was no group allocation.
Blinding	The study used a completely within-subject design where the condition would be randomly selected on a trial-by-trial basis. The experimenter was not aware of which condition the participant was completing on any trial, but was aware of the overall aims of the research.

Reporting for specific materials, systems and methods

We require information from authors about some types of materials, experimental systems and methods used in many studies. Here, indicate whether each material, system or method listed is relevant to your study. If you are not sure if a list item applies to your research, read the appropriate section before selecting a response.

Materials & experimental systems

n/a	Involved in the study	
☒	☐	Antibodies
☒	☐	Eukaryotic cell lines
☒	☐	Palaeontology
☒	☐	Animals and other organisms
☐	☒	Human research participants
☒	☐	Clinical data

Methods

n/a	Involved in the study	
☒	☐	ChIP-seq
☒	☐	Flow cytometry
☒	☐	MRI-based neuroimaging

Human research participants

Policy information about studies involving human research participants

Population characteristics	All participants were recruited from a paid participant pool administered by the School of Psychology at the University of Queensland. In Experiment 1, 22 participants (13 females, 9 males; median age 22 years; range 19-33 years) were recruited from a paid participant pool. In Experiment 2, 23 participants (14 females, 9 males; median age 23 years; range 19-33 years old were recruited from the same pool. The sample is representative from a young university student population which are commonly used to measure perception and attention in an easily-accessible group.
Recruitment	All participants were recruited from a paid participant pool administered by the School of Psychology at the University of Queensland and reimbursed at AUD$20/hr. The participants choose to participate based on a brief description of the study. It is unlikely this caused a significant bias in the research sample.
Ethics oversight	The University of Queensland Human Research Ethics Committee

Note that full information on the approval of the study protocol must also be provided in the manuscript.
