# Supplementary material for: Neural dynamics of the attentional blink revealed by encoding orientation selectivity during rapid visual presentation
Source: Nat Commun. 2020 Jan 23;11:434. doi: 10.1038/s41467-019-14107-z (PMC6978470; doi:10.1038/s41467-019-14107-z)
Supplement: Supplementary file 4 — Description of Additional Supplementary Files [file 41467_2019_14107_MOESM4_ESM.pdf]

Description of Additional Supplementary Files

File Name: Supplementary Movie 1
Description: Examples of two trials from the RSVP task.
